# Integration of Nutrient Sensing in Fish Hypothalamus

**DOI:** 10.3389/fnins.2021.653928

**Published:** 2021-02-26

**Authors:** José L. Soengas

**Affiliations:** Laboratorio de Fisioloxía Animal, Departamento de Bioloxía Funcional e Ciencias da Saúde, Facultade de Bioloxía and Centro de Investigación Mariña, Universidade de Vigo, Vigo, Spain

**Keywords:** fish, food intake regulation, nutrient sensors, transcription factors, hypothalamic integration

## Abstract

The knowledge regarding hypothalamic integration of metabolic and endocrine signaling resulting in regulation of food intake is scarce in fish. Available studies pointed to a network in which the activation of the nutrient-sensing (glucose, fatty acid, and amino acid) systems would result in AMP-activated protein kinase (AMPK) inhibition and activation of protein kinase B (Akt) and mechanistic target of rapamycin (mTOR). Changes in these signaling pathways would control phosphorylation of transcription factors cAMP response-element binding protein (CREB), forkhead box01 (FoxO1), and brain homeobox transcription factor (BSX) leading to food intake inhibition through changes in the expression of neuropeptide Y (NPY), agouti-related peptide (AgRP), pro-opio melanocortin (POMC), and cocaine and amphetamine-related transcript (CART). The present mini-review summarizes information on the topic and identifies gaps for future research.

## Introduction

Two neuronal populations in the mammalian hypothalamic arcuate nucleus respond to a rise in the levels of circulating metabolites (fatty acid, glucose, and amino acid) (Blouet and Schwartz, 2010; Morton et al., 2014). They respond with decreased expression of agouti-related peptide (AgRP) and neuropeptide Y (NPY) or increased expression of cocaine and amphetamine-related transcript (CART) and pro-opio melanocortin (POMC). These two populations inhibit each other and signal to higher-order neurons in other areas producing other neuropeptides, and food intake changes due to all these interactions.

In fish, AgRP/NPY and POMC/CART neurons are present in the ventral part of *nucleus lateralis tuberis* (NLTv), an analog of arcuate nucleus (Soengas et al., 2018). These neurons connect to other neuronal populations, both in hypothalamic and extra-hypothalamic locations, though their neuropeptide production is mostly unknown (Soengas et al., 2018). The regulation of energy expenditure and food intake relies on detection in vertebrate hypothalamus of changes in nutrient levels through different sensing mechanisms as demonstrated in mammals (Efeyan et al., 2015; Bruce et al., 2017) and fish (Soengas, 2014; Delgado et al., 2017; Soengas et al., 2018). Evidence obtained in rainbow trout (Polakof et al., 2009; Otero-Rodiño et al., 2019b) suggest a relationship between neuropeptide expression and nutrient sensing based on the simultaneous presence in hypothalamic areas of proteins involved in nutrient sensing, such as glucokinase (GCK), and the four neuropeptides. The activation of nutrient sensors enhances the anorexigenic potential (ratio between mRNA abundance of anorexigens and orexigens) through decreased expression of AgRP and NPY and increased expression of CART and POMC resulting in a decrease in food intake (Delgado et al., 2017; Soengas et al., 2018).

Studies carried out in rainbow trout (Librán-Pérez et al., 2012, 2013, 2014, 2015; Velasco et al., 2016a, 2020; Roy et al., 2020) demonstrated the presence in hypothalamus of fatty acid sensing mechanisms responsive to changes in the levels of long-chain fatty acids like oleate through carnitine palmitoyl transferase-1, mitocondrial ROS production inhibiting ATP-dependent inward rectified potassium channel (K^+^_*ATP*_), specific fatty acid receptors (FFAR), fatty acid translocase, and lipoprotein lipase. Evidence is also available in other species like grass carp (Li et al., 2016; Tian et al., 2017), Senegalese sole (Conde-Sieira et al., 2015), zebrafish (Liu et al., 2017), Chinese perch (Luo et al., 2020), and blunt snout bream (Dai et al., 2018). Mechanisms are similar to those characterized in mammals (Lipina et al., 2014; Magnan et al., 2015) though fish are also sensitive to polyunsaturated fatty acid like α-linolenate and medium-chain fatty acid like octanoate. Glucose is also sensed in fish hypothalamus. Evidence obtained suggested the presence in rainbow trout (Polakof et al., 2007a, b, 2008a, b, c) and Japanese flounder (Liu et al., 2019) of the canonical mechanism based on GCK, glucose facilitative transporter 2, and K^+^_*ATP*_. Evidence also supports mechanisms not dependent on GCK like those based on liver X receptor and sweet taste receptor (Otero-Rodiño et al., 2015, 2016, 2017; Balasubramanian et al., 2016). Leucine is the unique branched-chain amino acid (BCAA) whose levels are detected by mammalian hypothalamic amino acid sensing mechanisms (Efeyan et al., 2015; Heeley and Blouet, 2016). In fish, available evidence is restricted to rainbow trout (Comesaña et al., 2018a, b) and Chinese perch (Chen et al., 2021), suggesting the existence of mechanisms based on BCAA metabolism, glutamine metabolism, mechanistic target of rapamycin (mTOR), general control non-derepressible 2 kinase, and taste receptor signaling.

The connection between nutrient-sensing systems and the expression of AgRP/NPY and POMC/CART governing food intake is not clear. In mammals, several transcription factors could be involved (López et al., 2007; Diéguez et al., 2011) including brain homeobox transcription factor (BSX), phosphorylated cAMP response-element binding protein (CREB), and forkhead box01 (FoxO1). Changes in these transcription factors would respond to nutrient-sensing systems through mediation by different signaling pathways (Gao et al., 2013; Morton et al., 2014) including AMP-activated protein kinase (AMPK), mTOR, and protein kinase B (Akt). In the following sections, we will show available knowledge in fish regarding each of these pathways.

## Signaling Pathways

### AMPK

As an energy sensor, AMPK detects lowered cell energy status-eliciting mechanisms to restore energy balance (López, 2017). Thus, when levels of nutrients rise, a decrease occur in the levels and phosphorylation status of AMPK in mammalian hypothalamus, as demonstrated for glucose, leucine, and fatty acid (Diéguez et al., 2011; Fromentin et al., 2012; Oh et al., 2016).

In fish, several studies evaluated AMPK in brain (Zeng et al., 2016; Xu et al., 2018; Abernathy et al., 2019; Yang et al., 2019) but only a few addressed its role in the regulation of feed intake. Thus, in rainbow trout fed a lipid-enriched diet, a decrease occurred in phosphorylation status of hypothalamic AMPKα (Librán-Pérez et al., 2015). This is comparable with that observed in the same species (Kamalam et al., 2012) or in *Megalobrama amblycephala* (Xu et al., 2017) when fed a carbohydrate-enriched diet. Also in rainbow trout, raising levels of nutrients like oleate (Velasco et al., 2016a, 2017b; Blanco et al., 2020), octanoate (Velasco et al., 2017b), glucose (Otero-Rodiño et al., 2017), or β-hydroxybutyrate (Comesaña et al., 2019) also resulted in a decrease in phosphorylation status of AMPKα. The specificity of AMPKα response was supported by the disappearance of responses to oleate in the presence of the AMPKα inhibitor compound C (Velasco et al., 2017b). In contrast to the mammalian model, no changes in phosphorylation status of AMPKα occurred in rainbow trout hypothalamus in response to raised leucine levels (Comesaña et al., 2018a, b, 2020). Changes in AMPK also occurred in liver of fish species under different feeding status including a decrease in refed rainbow trout (Polakof et al., 2011) or an increase in food-deprived zebrafish (Craig and Moon, 2011). A role for hypothalamic AMPKα in food intake control is also supported by the increase observed in hypothalamus of food-deprived rainbow trout (Conde-Sieira et al., 2019). The involvement of AMPK is further supported by the decrease in phosphorylation status of AMPKα observed in rainbow trout hypothalamus under anorectic conditions like treatment with ceramide (Velasco et al., 2016b, 2017a), FFAR agonists (Velasco et al., 2020), or anorectic hormones like CCK (Velasco et al., 2019), GLP-1 (Velasco et al., 2019), or insulin (Blanco et al., 2020). Of the different isoforms of AMPKα, it seems that AMPKα2 is involved in feed intake regulation while AMPKα1 appears to modulate peripheral metabolism (Conde-Sieira et al., 2019).

### mTOR

Several studies demonstrate increased mTOR mRNA abundance and phosphorylation status in hypothalamus after a rise in the levels of leucine in mammals (Hu et al., 2016; Pena-Leon et al., 2020). In contrast, the effects of fatty acid or glucose on central mTOR are mostly unknown (André and Cota, 2012). However, the anorectic response induced by leptin treatment increase phosphorylation status of mTOR (Kwon et al., 2016) whereas the orexigenic response induced by ghrelin treatment (André and Cota, 2012) or food deprivation (Ferro Cavalcante et al., 2020) decreased mTOR.

In fish, mTOR was characterized in hypothalamus of several species including rainbow trout (Velasco et al., 2017b; Comesaña et al., 2018a; Blanco et al., 2020) and Japanese sea bass (Liang et al., 2019). In rainbow trout, mTOR levels and phosphorylation status responded with an increase to the presence of nutrients like oleate (Velasco et al., 2017b; Blanco et al., 2020), octanoate (Velasco et al., 2017b), glucose (Blanco et al., 2020), or leucine (Comesaña et al., 2018a, b). The presence of rapamycin blocked the response to fatty acids in hypothalamus (Velasco et al., 2017b) supporting the specificity of the response. Additional studies relate mTOR to food intake regulation in fish. Thus, increased mTOR occurred under anorectic conditions like feeding a lipid-enriched diet in rainbow trout (Librán-Pérez et al., 2015) and blunt snout bream (Dai et al., 2018) or different treatments in rainbow trout with PYY (Velasco et al., 2018), CCK (Velasco et al., 2019), insulin (Blanco et al., 2020), or ceramide (Velasco et al., 2017a). In other fish species, available information is limited. Thus, in Japanese sea bass, hypothalamic mTOR activation modulates POMC and NPY expression (Liang et al., 2019) whereas in cavefish, CCK treatment increased mTOR levels (Penney and Volkoff, 2014). In liver, mTOR phosphorylation status also changed under different feeding status. mTOR increases after a rise in levels of amino acids (Lansard et al., 2011; Wacyk et al., 2012; Liang et al., 2016; Skiba-Cassy et al., 2016), feeding lipid-enriched diets (Librán-Pérez et al., 2015; Zeng et al., 2016), or refeeding (Lansard et al., 2009; Wade et al., 2014) while a decrease occurred under food deprivation (Craig and Moon, 2011).

### Akt

In mammals, Akt levels and phosphorylation status in hypothalamus increased in response to a rise in the concentration of nutrients such as glucose (Chalmers et al., 2014), leucine (Hu et al., 2016), and β-hydroxybutyrate (Park et al., 2011). This response also occurred under anorectic situations in which hypothalamic POMC mRNA abundance increase (Kwon et al., 2016).

In fish, several evidence suggests the involvement of Akt in the hypothalamic mechanisms related to food intake control. Treatment with nutrients activates this signal through increased phosphorylation status, as demonstrated in rainbow trout for oleate (Blanco et al., 2020), octanoate (Velasco et al., 2017b), glucose (Otero-Rodiño et al., 2017), and leucine (Comesaña et al., 2018a). This activation also occurred under anorectic conditions such as treatment with ceramide (Velasco et al., 2016b, 2017a), insulin (Blanco et al., 2020), leptin (Gong et al., 2016), or FFAR agonists (Velasco et al., 2020). A comparable increase occurred in brain when fish were fed diets enriched in carbohydrates, as observed in zebrafish (Jörgens et al., 2015) and rainbow trout (Dai et al., 2014; Jin et al., 2014) or by feeding lipid-enriched diets as demonstrated in rainbow trout (Librán-Pérez et al., 2015) and blunt snout bream (Dai et al., 2018). The response of Akt to the rise in fatty acid levels disappeared in the presence of Akt inhibitor perifosine (Velasco et al., 2017b). Akt involvement in food intake regulation is also supported by the opposed response (decreased phosphorylation status) elicited by the orexigenic ghrelin treatment (Velasco et al., 2017a). In mammals, the activation of Akt signaling in the hypothalamus also resulted in changes in fatty acid metabolism due to the activation of sterol regulatory element-binding protein 1 (SREBP-1) and its target genes ATP citrate synthase (ACLY) and fatty acid synthase (FAS) (Kim et al., 2007). In rainbow trout hypothalamus, enhanced phosphorylation of Akt also occurred in parallel with mRNA abundance of *acly*, *fas*, and *srebp1* (Velasco et al., 2016b). Finally, besides central action, Akt is involved in peripheral responses to changes in feeding *status*. Thus, refeeding enhanced Akt phosphorylation in liver of barramundi (Wade et al., 2014) and rainbow trout (Lansard et al., 2009; Seiliez et al., 2011), and in muscle of Senegalese sole (Borges et al., 2014).

## Transcription Factors

### BSX

The transcription factor BSX interacts with CREB resulting in a parallel increase in the mRNA abundance of *Bsx, Npy*, and *Agrp* in mammalian hypothalamus (Nogueiras et al., 2008; Varela et al., 2011; Lee et al., 2016). Accordingly, *Bsx* decrease under anorectic conditions such as feeding a high-fat diet (Nogueiras et al., 2008) and increase under orexigenic conditions such as food deprivation (Nogueiras et al., 2008) or ghrelin treatment (Lage et al., 2010).

In fish, evidence regarding BSX role in hypothalamus is limited (Cremona et al., 2004; Schredelseker et al., 2020) with a few studies in rainbow trout related to food intake control. In this species, the exposure to oleate (Conde-Sieira et al., 2018) or glucose (Conde-Sieira et al., 2018; Blanco et al., 2020) reduced food intake in parallel with decreased BSX levels. There is no information available for BSX response in fish hypothalamus to the rise in amino acid levels. Other conditions known to decrease food intake in this species also decreased values for BSX as demonstrated by treatment with CCK (Velasco et al., 2019), GLP-1 (Velasco et al., 2019), or FFAR agonists (Velasco et al., 2020). No comparable studies assessed changes in *Bsx* mRNA expression under conditions of raised nutrient levels, not even in mammals. However, indirect evidence is available such as the effects of the treatment with the anorectic hormone leptin in rat resulting in decreased mRNA levels of *Bsx* in arcuate nucleus (Nogueiras et al., 2008) as well as in whole hypothalamus (Gao et al., 2011) in parallel with decreased food intake and *Npy* mRNA levels. In addition, situations in which an orexigenic response occurred (such as those elicited by ghrelin treatment or food deprivation) induced a rise in hypothalamic *Bsx* mRNA abundance (Nogueiras et al., 2008; Lage et al., 2010).

### CREB

cAMP response-element binding protein is another transcription factor hypothesized to be involved in the connection between brain metabolism and neuropeptides expression. Accordingly, in mammals, a decrease in CREB levels induced a decrease in mRNA abundance of *Npy* and *Agrp* leading to a decrease in food intake (Belgardt et al., 2009; Blanco de Morentin et al., 2011; Varela et al., 2011). CREB protein abundance decrease when food intake is inhibited enhancing anorexigenic potential through a decrease in mRNA levels of *Npy* and *Agrp* (Fukushima et al., 2015; Kwon et al., 2016) while levels increase under orexigenic situations such as ghrelin treatment (Lage et al., 2010) or food deprivation (Ren et al., 2013).

In fish, available information regarding CREB involvement in food intake regulation is restricted to rainbow trout. In this species, CREB phosphorylation status decreased in response to raised levels of oleate (Velasco et al., 2017b; Conde-Sieira et al., 2018), octanoate (Velasco et al., 2017b), glucose (Conde-Sieira et al., 2018; Otero-Rodiño et al., 2019a), or leucine (Comesaña et al., 2018a, b). CREB response to fatty acids was abolished by the presence of the CBP-CREB interaction inhibitor (Velasco et al., 2017b). Changes observed in CREB are comparable with those observed under other anorectic situations such as treatment with CCK o GLP-1 (Velasco et al., 2019). Moreover, increased levels of CREB occurred in zebrafish under the orexigenic conditions elicited by food deprivation (Craig and Moon, 2011).

### FoxO1

Forkhead box01 is likely involved in the relationship between metabolic changes in hypothalamus and the production of neuropeptides (Gross et al., 2009). Thus, in mammals, situations in which Fox01 increased resulted in an enhancement of *Agrp* mRNA values while those of *Pomc* decreased, changes favoring a decrease in food intake (Belgardt et al., 2009; Blanco de Morentin et al., 2011).

In fish, FoxO1 was characterized in brain in rainbow trout (Conde-Sieira et al., 2018) and turbot (Pan et al., 2019). Increased levels of nutrients enhanced its abundance and phosphorylation status as observed in rainbow trout for oleate (Conde-Sieira et al., 2018; Blanco et al., 2020), octanoate (Velasco et al., 2017b), and glucose (Conde-Sieira et al., 2018; Blanco et al., 2020). The specificity of FoxO1 response to raised levels of fatty acid was supported by its lack of response in the presence of the FoxO1 inhibitor AS1842856 (Velasco et al., 2017b). In contrast, FoxO1 does not appear to respond to changes in the levels of leucine (Comesaña et al., 2018a, b, 2020). Other anorectic conditions also resulted in increased FoxO1 in rainbow trout as observed after treatment with CCK (Velasco et al., 2019), GLP-1 (Velasco et al., 2019), insulin (Blanco et al., 2020), ceramide (Velasco et al., 2016b, 2017a), or FFAR agonists (Velasco et al., 2020). Central changes in FoxO1 are comparable with those occurring in peripheral tissues under different feeding status. Thus, in liver of rainbow trout orexigenic conditions like refeeding decreased FoxO1 levels (Dai et al., 2013) whereas in grass carp adipogenesis (a situation comparable with a rise in nutrient levels) increased FoxO1 in adipocytes (Sun et al., 2017). No prior studies in any other vertebrate species addressed the hypothalamic response of *Foxo1* to changes in nutrient levels. However, changes observed in fish would be comparable with those observed in mammalian hypothalamus under anorectic conditions like feeding a high-fat diet (Yuan et al., 2012) or treatment with insulin or leptin (Diéguez et al., 2011; Kwon et al., 2016). Akt activation is known to induce in mammals the phosphorylation of FoxO1 (Belgardt et al., 2009; Gross et al., 2009), which also result in enhanced CART and POMC expression (Kwon et al., 2016). In fish, a simultaneous rise in Akt and FoxO1 occurred in rainbow trout hypothalamus in response to anorectic treatments like oleate (Blanco et al., 2020), octanoate (Velasco et al., 2017b), or insulin (Blanco et al., 2020) but not leucine (Comesaña et al., 2018a). These results allow me to suggest a relationship between Akt and FoxO1 comparable with that suggested in mammals though restricted to several anorectic conditions.

## Interaction Nutrients-Hormones

AgRP/NPY and CART/POMC neurons involved in the integration of signals from nutrient sensors also have receptors for hormones like leptin, ghrelin, insulin, CCK, or GLP-1, among others (Blouet and Schwartz, 2010; Morton et al., 2014). The binding of these hormones to their receptors in hypothalamic neurons elicits changes in intracellular signals like AMPK and mTOR. Therefore, the final effect on food intake elicited by changes in neuropeptide expression would result from the interaction on signal transduction of both nutrients and hormones. However, this interaction is mostly unknown, with only some evidence available regarding the interactive effects of leptin or ghrelin on fatty acid sensing (López et al., 2007; Blanco de Morentin et al., 2011; Lockie et al., 2019).

In fish, only two studies carried out in rainbow trout demonstrated interactive effects in hypothalamus between nutrient-sensing mechanisms and hormones such as ghrelin and insulin. The presence of oleate counteracted ghrelin effects on AMPK (Velasco et al., 2016a). In the case of insulin (Blanco et al., 2020), its counteractive effects occurred in the presence of glucose for *bsx* and *mtor* mRNA abundance as well as in the presence of oleate for Akt phosphorylation status, and *foxo1* and *creb1* mRNA abundance. No other studies characterized in fish putative interactions in hypothalamus. However, in rainbow trout treatment with different hormones alter nutrient sensing mechanisms in hypothalamus (Conde-Sieira and Soengas, 2017) as demonstrated for glucosensing (leptin, insulin, ghrelin, nesfatin-1, CCK, and GLP-1) and fatty acid sensing (insulin, ghrelin, PYY, and nesfatin-1). Therefore, it is reasonable to hypothesize the existence of additional interactions through changes in cellular signaling and transcription factors.

## Conclusion and Perspectives

**FIGURE 1:**
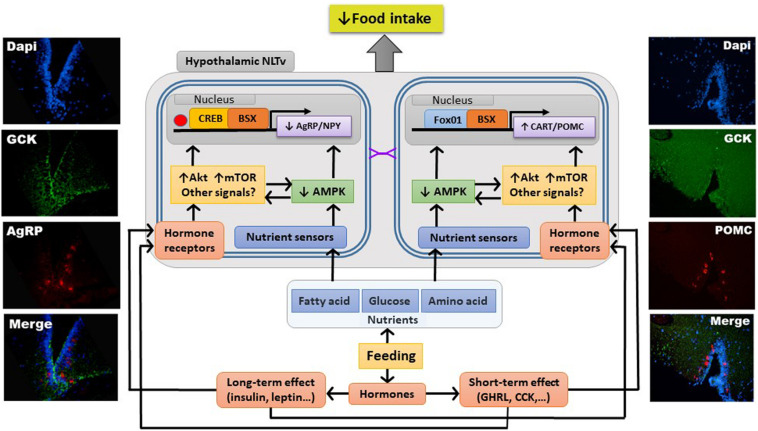
Schematic drawing with a model of processes involved in integration of nutrient-sensing information in fish hypothalamus resulting in homeostatic control of food intake. Left and right panels show pictures showing colocalization of a nutrient-sensing marker [glucokinase (GCK)] with AgRP (left) or POMC (right) in neurons (stained with DAPI) from rainbow trout hypothalamus. Purple symbols, synaptic connection; AgRP, agouti-related peptide; Akt, protein kinase B; AMPK, AMP-activated protein kinase; BSX, brain homeobox transcription factor; CART, cocaine- and amphetamine-related transcript; CCK, cholecystokinin; CREB, cAMP response-element binding protein; FoxO1, forkhead box protein O1; GHRL, ghrelin; mTOR, mechanistic target of rapamycin; NLTv, lateral tuber nucleus pars ventralis; NPY, neuropeptide Y; POMC, pro-opio melanocortin.

The knowledge available in fish about hypothalamic integration of information of metabolic and endocrine nature eliciting changes in expression of neuropeptides ultimately regulating food intake is limited (Delgado et al., 2017; Soengas et al., 2018). Studies in fish suggest the existence of a network similar in some aspects (but not in others) to that of mammals. In this network, the activation of the nutrient-sensing systems would result in the activation of Akt and mTOR as well as in the inhibition of AMPK. Changes in these signals would result in enhanced levels and phosphorylation status of FoxO1 while decreasing those of CREB and BSX. Finally, these changes in transcription factors would ultimately lead to inhibition of food intake inhibition through changes in neuropeptides (AgRP, NPY, POMC, and CART) expression, as observed in fish hypothalamus under anorectic conditions elicited by raised levels of nutrients. However, the precise mechanisms involved and their interaction with hormones still needs evaluation. A summary of available knowledge is shown in Figure 1.

## Author Contributions

The author confirms being the sole contributor of this work and has approved it for publication.

## Conflict of Interest

The author declares that the research was conducted in the absence of any commercial or financial relationships that could be construed as a potential conflict of interest.
